# Identification and Characterization of *NBS* Resistance Genes in *Akebia trifoliata*

**DOI:** 10.3389/fpls.2021.758559

**Published:** 2021-10-29

**Authors:** Xiaojiao Yu, Shengfu Zhong, Huai Yang, Chen Chen, Wei Chen, Hao Yang, Ju Guan, Peng Fu, Feiquan Tan, Tianheng Ren, Jinliang Shen, Min Zhang, Peigao Luo

**Affiliations:** ^1^Provincial Key Laboratory for Plant Genetics and Breeding, Chengdu, China; ^2^College of Agronomy, Sichuan Agricultural University, Chengdu, China; ^3^Sichuan Akebia trifoliata Biotechnology Co., Ltd., Chengdu, China; ^4^College of Forestry, Sichuan Agricultural University, Chengdu, China

**Keywords:** *Akebia trifoliata*, *NBS* genes, gene duplication, pathogen, genome-wide analysis

## Abstract

*Akebia trifoliata* is an important multiuse perennial plant that often suffers attacks from various pathogens due to its long growth cycle, seriously affecting its commercial value. The absence of research on the resistance (*R*) genes of *A. trifoliata* has greatly limited progress in the breeding of resistant varieties. Genes encoding proteins containing nucleotide binding sites (NBSs) and C-terminal leucine-rich repeats (LRRs), the largest family of plant resistance (*R*) genes, are vital for plant disease resistance. A comprehensive genome-wide analysis showed that there were only 73 *NBS* genes in the *A. trifoliata* genome, including three main subfamilies (50 *coiled coil* (*CC*)-*NBS-LRR* (*CNL*), 19 *Toll/interleukin-1 receptor* (*TIR*)-*NBS-LRR* (*TNL*) and four *resistance to powdery mildew8* (*RPW8*)-*NBS*-*LRR* (*RNL*) genes). Additionally, 64 mapped *NBS* candidates were unevenly distributed on 14 chromosomes, most of which were assigned to the chromosome ends; 41 of these genes were located in clusters, and the remaining 23 genes were singletons. Both the *CNLs* and *TNLs* were further divided into four subgroups, and the *CNLs* had fewer exons than the *TNLs*. Structurally, all eight previously reported conserved motifs were identified in the NBS domains, and both their order and their amino acid sequences exhibited high conservation. Evolutionarily, tandem and dispersed duplications were shown to be the two main forces responsible for *NBS* expansion, producing 33 and 29 genes, respectively. A transcriptome analysis of three fruit tissues at four developmental stages showed that *NBS* genes were generally expressed at low levels, while a few of these genes showed relatively high expression during later development in rind tissues. Overall, this research is the first to identify and characterize *A. trifoliata NBS* genes and is valuable for both the development of new resistant cultivars and the study of molecular mechanisms of resistance.

## Introduction

*Akebia trifoliata* (Thunb.) Koidz, belonging to the family Lardizabalaceae (Liu et al., 2007), presents great potential for use as a fruit and oil crop and a medicinal and ornamental plant (Li L. et al., 2010; Chen et al., 2017). After extensive artificial cultivation, wild plants will inevitably suffer from various diseases, such as kiwifruit bacterial canker (Scortichini et al., 2012), sweet potato root rot disease (Ma et al., 2020) and apple fruit anthracnose (Zhou and Zhou, 2017); this is especially true for perennials, such as black cottonwood, which often suffer attacks from various pathogens or herbivores before reaching the reproductive stage because of their relatively long life cycle (Tuskan et al., 2006). Likewise, *A. trifoliata* is a typical perennial that is attacked by various pathogens, such as *Alternaria tenuissima*, causing leaf spot (Ye et al., 2013; Zhang et al., 2015), *Colletotrichum gloeosporioides*, causing leaf anthracnose (Cheng et al., 2020; Pan et al., 2020), *Nigrospora sphaerica*, causing fruit shrinkage (Hong et al., 2021), and *Oidium subgenus Pseudoidium*, causing powdery mildew (Garibaldi et al., 2004), which has resulted in large yield losses and significantly reduced the economic value of the species.

During the arms race between hosts and their pathogens, great numbers of variations arise in pathogens due to their short growth cycle and simple genomic sequences, requiring plants (especially perennials) to evolve a set of sophisticated and effective defense systems to combat them (Tuskan et al., 2006). Beyond the first line of defense against pathogens imposed by the thickened cell wall, plants usually build the second defense system by producing resistance proteins, mainly encoded by *NBS* genes, to resist invading pathogens after the failure of the cell wall defense system (Andersen et al., 2018). By directly or indirectly recognizing pathogen-secreted effectors, these proteins confer resistance to various pathogens, including fungi, bacteria and viruses, by initiating a series of defense responses, such as hypersensitive responses, activating signaling pathways and consequently inhibiting the plant infection process (Andersen et al., 2018).

The *NBS* gene family, encoding proteins containing nucleotide binding sites (NBSs) and C-terminal leucine-rich repeats (LRRs), is the largest family of plant resistance (*R*) genes, accounting for over 60% of detected and cloned *R* genes in all plant species (Kourelis and van der Hoorn, 2018). The NBS domain can bind ATP/GTP, resulting in phosphorylation to transmit disease resistance signals downstream, which plays a key role in combating almost all pathogens (Bent, 1996). According to the N-terminal structure of *NBS* genes, scientists have commonly divided these genes into three subfamilies: (*TIR*)-*NBS-LRR* (*TNL*) genes, containing TIR domains with homology to Toll/interleukin-1 receptors, and (*CC*)-*NBS-LRR* (*CNL*) and (*RPW8*)-*NBS*-*LRR* (*RNL*), characterized by CC and RPW8 domains, respectively (Xiao et al., 2001; Collier and Moffett, 2009; Wang et al., 2013). The *RNL* clade is composed of two different lineages: the *Nicotiana benthamiana* N-required gene 1 (*NRG1*) and *Arabidopsis* activated disease resistance gene 1 (*ADR1*) lineages. To clearly indicate the absence of the TIR domain compared with *TNLs*, we refer to *CNLs* and *RNLs* as the *non-TNL* (*nTNL*) subclass. Generally, TNL and CNL proteins are mainly responsible for recognizing specific pathogens, while RNL proteins may play an auxiliary role in downstream defense signal transduction.

With the completion of the sequencing of many plant genomes in succession, genome-wide analysis has increasingly become an important tool for studying the genetic diversity and evolution of *NBS* resistance genes (Meyers et al., 2003; Xue et al., 2019; Zhang et al., 2020). The number of *NBS* genes in a plant genome ranges from dozens to more than 2,000 (Shao et al., 2019; Andersen et al., 2020), leading to the discussion of whether the *NBS* gene number is related to genome size and, if so, whether the relationship is positive or not (Liu et al., 2014; Zhang et al., 2016; Goyal et al., 2020). In addition, the composition of the three subclasses is usually not the same or is even highly lopsided. For instance, *Dioscorea rotundata* possesses 166 *CNLs*, only one *RNL* and no *TNLs* (Zhang et al., 2020), and *Brassica napus* contains 461 *TNLs*, 180 *CNLs* and no *RNLs* (Alamery et al., 2018), suggesting a major discrepancy between the different subclasses. The differences in the composition of *NBS* genes are considered to be responsible for the diversity and specificity of resistance to various pathogens (Jupe et al., 2012). *NBS* genes generally arise via various types of duplications, mainly including tandem and dispersed duplications, which have a critical influence on the different arrangements of *NBS* genes, such as the occurrence of singletons or clustered loci on chromosomes (Meyers et al., 2003; Leister, 2004).

The exploration of the characteristics of *NBS* genes usually involves phylogenetic and structural analyses in diverse plant species, which has greatly accelerated the identification and utilization of functional *R* genes. Although there has been no research progress on *A. trifoliata NBS* resistance genes to date, the available genome and transcriptome data of *A. trifoliata* not only enrich the genomic resources available for this species but also create favorable conditions for the systematic study of *NBS* genes at the genomic level. In this study, we comprehensively illustrated the *NBS* gene profile of *A. trifoliata* using multiple analysis tools. A collection of *R* gene resources valuable in production were screened, providing meaningful data for further identifying functional *R* genes and accelerating the future breeding of resistant cultivars of *A. trifoliata*.

## Materials and Methods

### Data Used in This Study

The *A. trifoliata* genome sequence, annotation files and RNA-seq data (accession IDs: SAMN16551931--33, young stage of rind, flesh and seed; SAMN16551934--36, enlargement stage; SAMN16551937--39, coloring stage; SAMN16551940--42, mature stage) were downloaded from the National Center for Biotechnology Information (NCBI) database under BioProjectID PRJNA671772,^1^ which was assembled and uploaded by our group. Reference genes with known resistance functions were also retrieved from NCBI protein database, accessions as listed in Supplementary Table 4.

### Identification and Classification of *NBS* Genes

To identify *A. trifoliata* homologs of plant *NBS* genes, we first performed a BLASTP analysis in NCBI^2^ to search NBS proteins with the NB-ARC domain query (accession: PF00931), and then the protein sequences were input to a hidden Markov model (HMM)^3^ for scanning using the HMM profile of the NB-ARC domain as a query. Both of the *E*-values were set as 1.0. We further merged all of the candidate genes obtained from the above two databases and removed the redundant genes. Finally, we analyzed the non-redundant genes against the Pfam database^4^ to further verify the presence of the NBS domain according to an *E*-value of 10^––4^ and to eliminate the genes without a conserved NBS domain. To classify the NBS genes, all of the identified NBS sequences were further analyzed using the NCBI Conserved Domain Database^5^ to determine the existence of TIR (accession: PF01582), RPW8 (accession: PF05659) and LRR (accession: PF08191) domains, whereas CC domains were identified by using Coiledcoil^6^ with a threshold value of 0.5 because these domains cannot always be identified by Pfam searches (Lozano et al., 2015).

### Gene Structure and Conserved Motif Analysis

The general feature format (GFF3) file of the *A. trifoliata* genomic annotation file was used to retrieve the *NBS* gene structure and exon information. For further analysis of the conserved domain composition of the *NBS* gene family, we predicted the conserved motifs in the NBS domains with the MEME Suite^7^. The motif count was set to 10 with motif width lengths ranging from 6 to 50 amino acids, and all other parameters were set to the defaults as previously described (Nepal et al., 2017). Then, TBtools^8^ was used to visualize the positions of motif and exon structures in *NBS* genes. The bivariate correlations between the characteristics and multiple comparisons thereof in the three subfamilies were performed using SPSS software (Dudley et al., 2004).

### Chromosomal Distribution of *NBS* Genes

The *A. trifoliata* genome was assembled into 16 chromosomes, and the GFF3 file was used to investigate the chromosome-wide distribution of *NBS* candidates and to map chromosomal physical positions. To identify the numbers of *NBS* gene clusters on chromosomes, we utilized sliding window analysis assuming a window size of 250 kb (Ameline-Torregrosa et al., 2008).

### Phylogenetic and Ka/Ks Analysis

To explore the underlying evolutionary history among *NBS* family members, ClustalW was used to perform multiple sequence alignments among all protein sequences with conserved NBS domains. Then, the result was manually corrected by removing gaps and sequences that were too short or less similar in MEGA X (Kumar et al., 2018) to reduce “noisy characters.” We constructed a phylogenetic tree using IQ-TREE with the maximum likelihood method (Nguyen et al., 2015), selected the best-fit model using ModelFinder (Kalyaanamoorthy et al., 2017), and evaluated branch support values via UFBoot2 tests (Hoang et al., 2018). A TNL tree consisting of 19 identified *TNLs* of *A. trifoliata* and 19 reference *TNLs* from other species and a CNL tree including 48 *CNLs* and four *RNLs* of *A. trifoliata* and 38 reference *CNLs* and two reference *RNLs* from other species (Supplementary Table 4) were reconstructed. All trees were rerooted using the NBS domain of human apoptotic protease-activating factor-1 (*APAF-1*), and the results were visualized with Figtree.^9^ To determine whether the *A. trifoliata NBS* genes were subject to selection pressure, the nucleotide coding sequences (CDSs) of each subfamily were aligned by using MEGA X. Then, KaKs_Calculator 2.0 was used to calculate the non-synonymous substitution to synonymous substitution (Ka/Ks) ratio for each orthologous *NBS* gene pair of *A. triloliata* genome (Wang et al., 2010), and ratios of > 1, = 1 and < 1 indicated positive selection, neutral evolution and purifying selection, respectively (Hurst, 2002).

### Duplication Analysis of *NBS* Genes

For synteny analysis, pairwise all-against-all BLAST searches were applied to the *A. trifoliata* NBS proteins (Xue et al., 2019). The results and GFF3 files were then submitted to MCScanX for gene duplication type detection (Wang et al., 2012).

### Expression Analysis of *NBS* Genes of *A. trifoliata* in Various Fruit Tissues

To analyze the expression pattern of *A. trifoliata NBS* genes in different tissues (flesh, rind and seeds) at different developmental stages (young, enlargement, coloring and maturity), BLAST in HISAT2 software was first used to align the RNA-seq data with the *A. trifoliata* genome under default settings. Then, we used SAMtools to compress the results into BAM format and extracted the FPKM values representing the expression of each gene in all samples according to previously described methods (Li et al., 2009; Kim et al., 2019). Finally, to display *NBS* gene expression levels, the transcriptome data were submitted to TBtools to generate a heatmap (Chen et al., 2020).

## Results

### Identification and Classification of *NBS* Genes in the *A. trifoliata* Genome

A total of 73 non-redundant *NBS* genes, accounting for only 0.30% of the 24,138 annotated genes, were identified in the *A. trifoliata* genome (see details in “Materials and Methods”). The percentage of *NBS* genes in *A. trifoliata* was slightly higher than those reported in *Carica papaya* and *Setaria italica* (0.22 and 0.27%, respectively) (Porter et al., 2009; Zhao et al., 2016), while it was obviously lower than those in most other plant species (Table 1). The 73 NBS protein sequences were classified into three groups (50 *CNL*s, 19 *TNL*s, and 4 *RNL*s) and nine subgroups according to the existence of CC, TIR and RPW8 domains, which are summarized in Table 2 and Supplementary Table 1. The *CNL* group was further divided into four subgroups: 31 *CNLs* containing a complete set of CC, NBS and LRR domains, 13 *CN_*CC*_s* lacking the LRR domain, four *NL_*CC*_s* lacking the CC domain, and two *N_*CC*_s* lacking both CC and LRR domains. Likewise, the *TNL* group consisted of five *TNLs* with the complete set of TIR, NBS, and LRR domains, 10 *NL_*TIR*_s* lacking the TIR domain, one *TN*_*TIR*_ lacking the LRR domain and three *N_*TIR*_s* lacking both TIR and LRR domains. In contrast, all four *RNLs* exhibited the complete set of RPW8, NBS and LRR domains and could not be further subdivided. The results showed that there were abundant variations in the domains of *A. trifoliata NBS* genes, although the number of *NBS* genes was lower than in many species, with exceptions including *C. papaya* and *S. italica*.

**TABLE 1:** The numbers of predicted ***NBS*** genes in sequenced plant genomes.

Species	*NBS* gene types			Total *NBS* genes	Predicted proteins	Proportion of *NBS* genes	Genome size (Mb)
	
	*CNL*	*TNL*	*RNL*				
*Aquilegia caerulea*	231	1	1	233	41,063	0.57%	306
*Nymphaea colorata*	234	115	11	360	31,589	1.14%	409
*Akebia trifoliata*	50	19	4	73	24,138	0.30%	645
*Amborella trichopoda*	84	25	1	110	31,494	0.35%	706
*Arabidopsis thaliana*	55	94	−	149	38,311	0.39%	125
*Carica papaya*	33	20	1	54	24,742	0.22%	372
*Populus trichocarpa*	236	123	−	359	41,444	0.87%	410
*Vitis vinifera*	241	111	−	352	29,585	1.19%	505
*Solanum lycopersicum*	222	31	2	255	34,727	0.73%	739
*Oryza sativa* L.	535	0	−	535	37,544	1.42%	389
*Dioscorea rotundata*	166	0	1	167	26,198	0.64%	594
*Setaria italica*	96	0	−	96	35,844	0.27%	406
*Triticum aestivum* L.	2148	5	3	2151	107,891	1.99%	15,770
Correlations between genome size and *NBS* gene number							0.96**^a^
							−0.14^b^

The letter “a” represents a correlation in all 13 species, while “b” indicates a correlation in all species except for Ta. “**” indicates P-values < 0.01, the same below.

**TABLE 2:** Groups of *NBS* resistance genes in the *A*. *trifoliata* genome.

Type	Predicted protein domain	Num.	Gene length (exon number)			
	
			Min	Max	Mean	Bivariate correlations
**CNL subgroup**		**50**	596 (1)	27583 (15)	6036.88^a^ (3.24)^a^	0.59**
CC-NBS-LRR (CNL)	CC, NBS, LRR	31				
CC-NBS (CN)	CC, NBS	13				
NBS-LRR (NL)	NBS, LRR	4				
NBS (N)	NBS	2				
**TNL subgroup**		**19**	1211 (1)	22071 (10)	7486.58^a^ (3.63)^a^	0.55*
TIR-NBS-LRR (TNL)	TIR, NBS, LRR	5				
TIR-NBS (TN)	TIR, NBS	1				
NBS-LRR(NL)	NBS, LRR	10				
NBS (N)	NBS	3				
**RNL subgroup**		**4**	3932 (2)	14166 (6)	8904.25^a^ (4.50)^a^	−0.12
RPW8-NBS-LRR (RNL)	RPW8, NBS, LRR	4				
Total		73			6571.32 (3.41)	0.57**

*The letter “a” indicates no significant difference in multiple comparisons.* Num., number; Min, minimum; Max, maximum. “*” indicates P-values < 0.05 and > 0.01, and “**” indicates P-values < 0.01, the same below.

### Gene Structure and Conserved Motif Analysis

**FIGURE 1:**
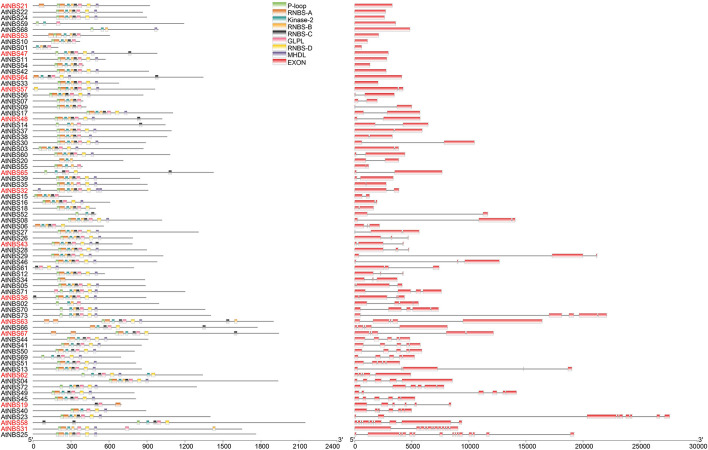
Gene structure and motif analysis of predicted *NBS* genes. Conserved motif analysis in the NBS domain was performed using the MEME Suite based on protein sequences. In total, eight conserved motifs were identified, and each motif is depicted with boxes of different colors. Their logos are shown in Supplementary Table 2. A gene name in red font indicates that the gene shows differences in motif order in the NBS domain. The intron/exon organization of *NBS* genes is represented here, and exon numbers increase gradually from the top to bottom; red boxes depicting exons are separated by introns with thin lines.

To outline the difference in gene structure between the different *NBS* subfamilies within *A*. *trifoliata*, the comparison analysis of exon number, gene length and both composition and order of the conserved motif was further executed. The results show that the exon number of the 73 *NBS* genes ranged from 1 to 15, with a total of 249 exons and a mean of 3.41 exons (Figure 1 and Supplementary Table 1). Among these genes, 14 (9 *CNL*s and 5 *TNL*s) had only one exon, and the number of genes with more than eight exons was 5. Although *AtNBS25* (*coiled coil* (*CC*)-*NBS-LRR* (*CNL*)) had the most exons, with 15, 36 (72.0%) of the 50 *CNLs* had fewer than 3 exons, while 11 (57.9%) of the 19 *TNLs* had more than 3 exons. We found that both the mean exon numbers and gene length of the *RNLs* (4.50 and 8904.25 bp) were greater than those of the *CNLs* (3.24 and 6036.88 bp) and *TNLs* (3.63 and 7486.58 bp), although there was no significant relationship between exon number and gene length found in multiple comparisons among the three subfamilies at the *P* = 0.05 level (Table 2). Further analysis showed that the relationship between exon number and gene length was significant at the *P* = 0.05 level among the 73 *NBS* genes (*r* = 0.57), the 50 *CNLs* (*r* = 0.59) and the 19 *TNLs* (*r* = 0.55), while it was not significant for the 4 *RNLs* (*r* = −0.12) (Table 2), which meant that the exon number per gene would be positively related to gene length to some degree.

MEME analysis showed that eight conserved motifs (P-loop, RNBS-A, Kinase-2, RNBS-B, RNBS-C, GLPL, RNBS-D, and MHDL, with the common arrangement order) that have been reported in other species, such as *Arabidopsis* (Meyers et al., 2003; Zhou et al., 2004), existed in various *NBS* genes of the *A*. *trifoliata* genome (Figure 1 and Supplementary Table 2), among which the P-loop (distributed in 66 genes), GLPL (in 68 genes) and kinase-2 (in 70 genes) were the most common motifs. While 57 of the 73 *NBS* genes showing the conserved order of the eight motifs, the remaining 16 genes showed a slight change in the motif order, among which 14 genes exhibited the conserved core motif order flanked by single or multiple repeated motifs on one or two sides and only 2 genes (*AtNBS19* and *AtNBS63*) showed a change in the actual core order (Figure 1 and Supplementary Table 2). In addition, we found that the last residue of the kinase-2 motif was W (tryptophan) in 48 (88.9%) of the 54 *non-TNL* genes, while it was D (aspartate) in 13 (68.4%) of the 19 *TNL*s (Supplementary Table 3). Thus, it was inferred that the *NBS* gene type could be distinguished by the last conserved codon of the kinase-2 motif.

### Chromosomal Distribution of *NBS* Genes in *A. trifoliata*

**FIGURE 2:**
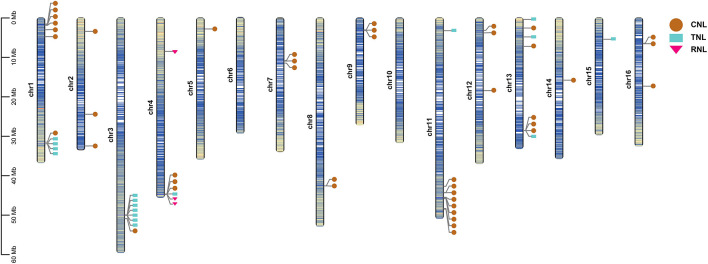
Chromosomal distribution of *NBS* genes in *A. trifoliata*. The relative mapping positions of 64 identified *NBS* genes are shown on the chromosomes. *CNL* genes are represented by brown circles, while *TNL* and *RNL* genes are represented by blue rectangles and pink triangles, respectively. The shade of the chromosome color represents gene density, where the bluer the color, the higher the density, and the yellower the color, the lower the density.

The physical locations of the 73 identified *NBS* genes were mapped on the 16 assembled chromosomes of the *A. trifoliata* genome by using TBtools. A total of 64 mapped genes (44 *CNL*s, 17 *TNL*s, and 3 *RNL*s) were unevenly distributed on 14 of the 16 chromosomes and mostly mapped to the regions near chromosome ends (Figure 2), whereas the remaining nine genes were excluded due to their locations in unassembled contigs. Chromosomes 1, 3, 11, and 13 contained more than seven *NBS* genes, while chromosomes 5, 14, 15, and 16 contained only one NBS gene (Figure 2). In addition, *CNLs* were widely distributed on 14 chromosomes, *TNLs* were distributed on six chromosomes, and all three *RNL*s were located on chromosome 4. There was obviously no significant correlation between *NBS* gene number and chromosome length.

According to the definition of gene clusters, the genes on the chromosomes were divided into 35 loci, including 23 singletons and 12 gene clusters (Supplementary Table 1), and 41 (64.1%) of 64 mapped *NBS* genes were distributed into 12 clusters, with a mean of 3.42 genes per cluster. The 12 defined gene clusters were assigned to 9 chromosomes: five clusters of loci had only two adjacent genes, 14 (on chromosome 7), 16 (on chromosome 8), 20 and 23 (on chromosome 11), and 34 (on chromosome 16); two clusters of loci included three genes, 17 and 21 (on chromosomes 9 and 11, respectively); three clusters of loci contained four genes, 1 and 5 (on chromosome 1) and 30 (on chromosome 13); one cluster of loci included six genes, 12 (on chromosome 4); and the remaining cluster of loci included seven genes (the greatest number), 9 (on chromosome 3).

### Ka/Ks and Phylogenetic Analysis

To differentiate both the type and the strength of natural selection between different *NBS* types, the Ka/Ks values of all orthologous *A. trifoliata NBS* genes were also calculated because it is usually as an informative indicator of natural selection during evolution progress. The results showed that all *RNL*s were excluded according to the lower limit for orthologs, and the ratios of all orthologous *NBS* genes were far less than 1 (Supplementary Table 5), which indicated that the *A. trifoliata NBS* genes have mainly experienced purifying selection during evolution (i.e., removing harmful mutations and maintaining protein conservation). The average Ka/Ks ratio of the *CNL*s was 0.31, which was significantly lower than that (0.42) of the *TNL*s at the *p* = 0.01 level.

**FIGURE 3:**
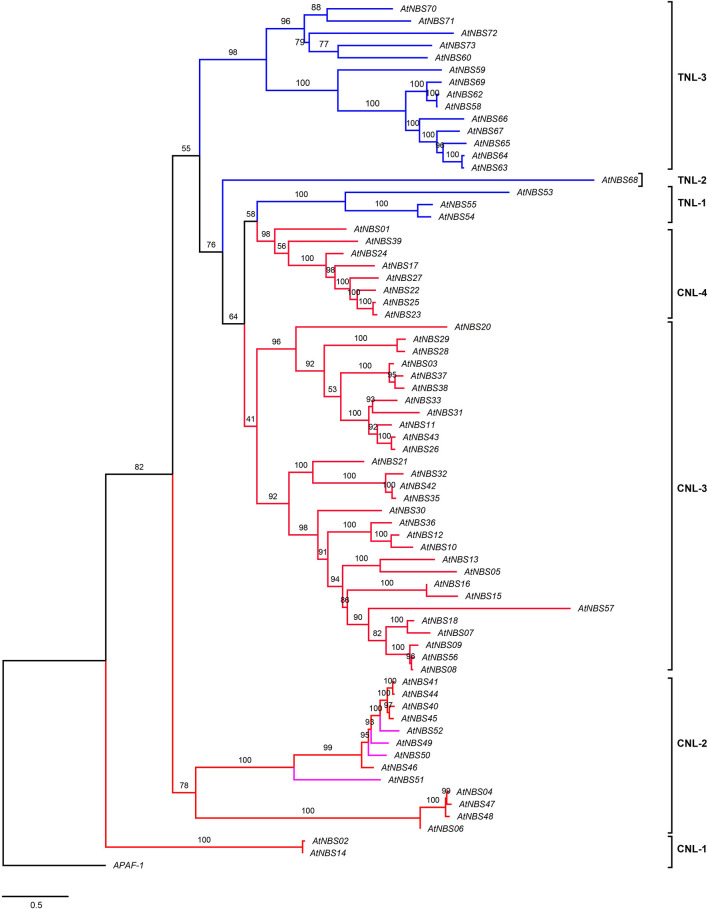
Phylogeny of *NBS* genes in *A. trifoliata*. The maximum likelihood tree was constructed using 70 NBS domains, both here and below. Percent bootstrap values (1000 iterations) are indicated in every branch. Blue, red, and pink lines correspond to the TIR, CC, and RPW8 clades, respectively. *APAF-1* was used as an outgroup. The scale bar indicates the genetic distance.

The evolutionary relationships among all the identified *NBS* candidates were inferred from the phylogenetic tree by using the NB-ARC domains, except for *AtNBS19*, *AtNBS34* (*CNL*), and *AtNBS61* (*TNL*) because of their shorter or less similar sequences. They were divided into two broad branches, the TNLs and CNLs (Figure 3). The TNL branch included three subgroups, TNL-1, TNL-2, and TNL-3, with 18 *TNL* genes, while the CNL branch contained four main subgroups, CNL-1, CNL-2, CNL-3, and CNL-4, with 52 genes, consisting of 48 *CNL*s and 4 *RNL*s. In addition, all 4 *RNL*s were clustered into the CNL-2 subgroup.

**FIGURE 4:**
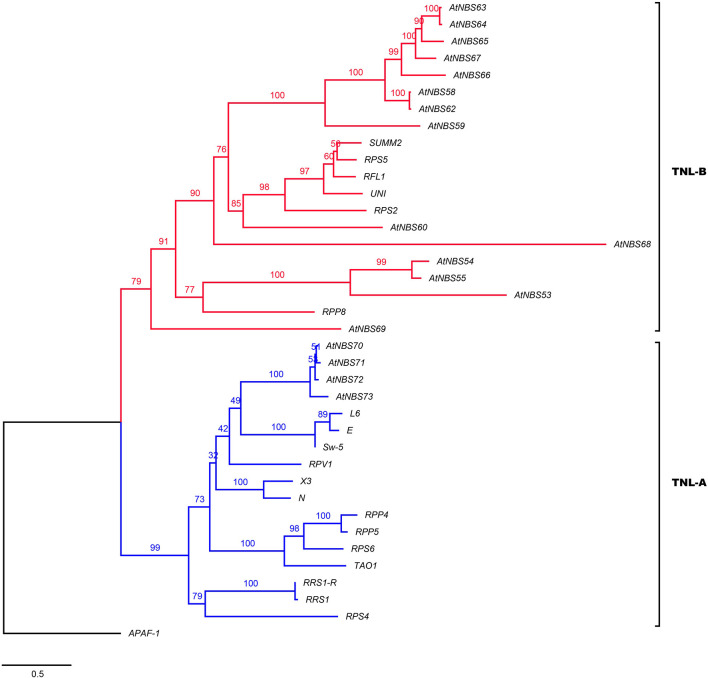
Phylogeny of the *TNL* subclass of *A. trifoliata* NBS proteins with functional resistance genes from other species. Blue and red represent the TNL-A and TNL-B clades.

**FIGURE 5:**
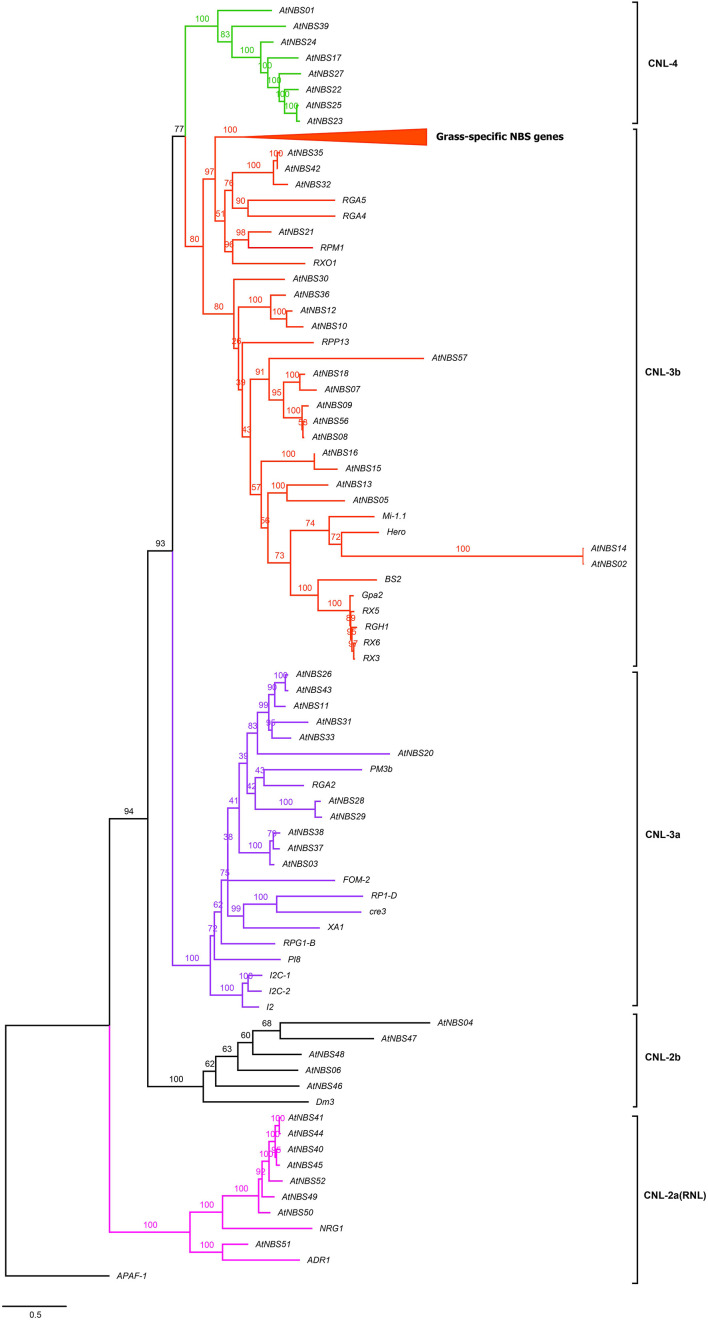
Reconstructed tree of the *CNL* subclass of *A. trifoliata* NBS proteins. Pink, black, purple, red and green represent the CNL-2a (RNL), CNL-2b, CNL-3a, CNL-3b, and CNL-4 clades, respectively. The top clade in the CNL-3b clade is compressed, and the complete tree is shown in Supplementary Figure 1 because it clusters *R* genes that are specific to grasses and do not group together with any *A. trifoliata NBS* candidates. Members of this clade include *Pi-ta* and *Pik-1/2* from *O. sativa* L. and *Mla1*, *MLA12* and *RPG5* from *Hordeum vulgare* L.

To identify close functional homologs for each *NBS* candidate, TNL and CNL trees were separately reconstructed by adding some clearly functional *NBS* genes: 19 *TNLs*, such as *RPS2* (*Arabidopsis thaliana*) (Bent et al., 1994; Figure 4), 38 *CNL*s, such as *Pi-ta* (*Oryza. sativa*) (Huang et al., 2008), and two *RNL*s, including *ADR1* (*A. thaliana*) (Collier et al., 2011; Figure 5), as references. The TNL tree was redivided into two subgroups: TNL-A and TNL-B (Figure 4). Four *TNL*s of *A. trifoliata* and 14 well-known functional *TNL*s that mainly confer resistance to fungal rust and spot diseases in species such as *Nicotiana tabacum* (*N*) and *Solanum lycopersicum* (*E*) were clustered into TNL-A, while TNL-B contained 14 *TNL*s of the *A. trifoliata* genome and five *TNL* reference genes that confer resistance to bacterial (*RPS5/2*, *RFL1* and *UNI* of *A. thaliana*) or fungal (*SUMM2* of *Cocos nucifera*) root rot diseases.

The branches in the CNL tree were classified into the CNL-2a/b, CNL-3a/b, and CNL-4 clades (Figure 5). Four *CNLs* and four *RNLs* from *A. trifoliata* and two *RNL* reference genes (*ADR1* and *NRG1*) clustered into clade CNL-2a (RNL). Clade CNL-2b contained five identified *CNLs* and one reference gene, *Dm3*, conferring resistance to downy mildew in *Lactuca sativa* L. In CNL-3a among 11 candidates and 11 reference genes, the reference genes preferentially clustered separately from *CNL* candidates. Although the powdery mildew resistance gene *PM3b* and the late blight resistance gene *RGA2* clustered with *A. trifoliata* genes, the bootstrap support was rather low. Although 20 candidates and 24 reference genes were included the same large CNL-3b clade, they still exhibited two relatively distinct branches because of many reference genes from grass families (Figure 5 and Supplementary Figure 1), which could be explained in terms of the more versatile reference *CNL* genes after the loss of *TNLs* in monocots (Li J. et al., 2010). Interestingly, only *A. trifoliata* genes clustered in CNL-4, as shown in Figure 3.

### Duplication Analysis of *NBS* Genes

The different duplication types were detected by MCscanX, and the output results showed that 33 (45.2%) of the 73 *NBS* genes were duplicated by tandem duplication. These genes were mainly distributed in clusters on chromosomes 1, 3, 4, 11, and 13. A total of 29 genes (39.7%) resulted from the dispersal of duplications of individual or small groups of genes to unlinked loci; 9 (12.3%) were produced by proximal duplication; and only 2 (2.7%) arose through whole-genome duplication (WGD) (Supplementary Table 1). In the *CNL* subfamily, 74.0% of the genes were derived from tandem and dispersed duplications; similarly, among the *TNLs* and *RNLs*, 84.2 and 50.0% of the genes, respectively, arose from the above two types of duplications.

### Differential Expression Analysis of *NBS* Genes of *A. trifoliata* in Various Fruit Tissues

**FIGURE 6:**
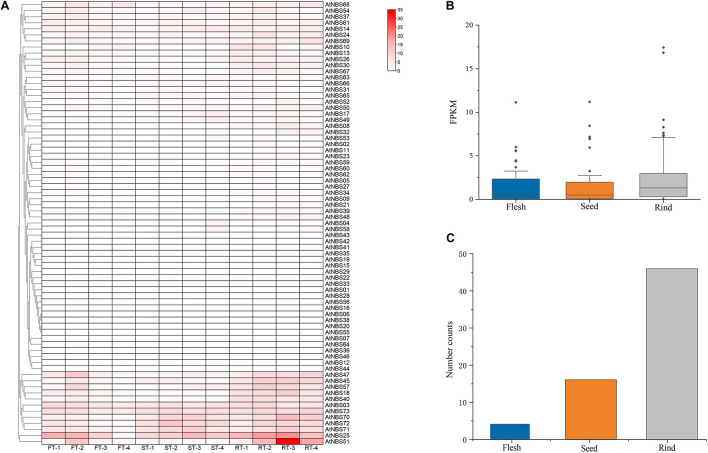
Expression profile of *A. trifoliata NBS* genes. **(A)** Expression heat map of 73 *NBS* genes in three different fruit tissues at four developmental stages. **(B)** Mean expression of 73 *NBS* genes in the three tissues. **(C)** Quantitative distribution of the highest expression levels of *NBS* genes in tissues.

To test the general function of the 73 *NBS* genes by expressing analysis, the RNA data of different tissues (flesh, rind and seeds) at four developmental stages (young, enlargement, coloring and maturity) were downloaded from NCBI BioSample^10^. The results of expression analysis showed that there were 66 genes expressed at detectable levels, most of which showed a very low expression level in all three fruits tissues at all four stages of development (Figure 6A). Only *AtNBS51*, with an FPKM value of more than 30, was identified as showing intermediate or high expression, while there were 27 genes with expression levels ranging from 3 to 30 FPKM categorized as genes with low expression. Interestingly, 26 of the 28 genes (16 *CNL*, 9 *TNL*, 3 *RNL*) were mainly expressed in rind, and 10 of them were increasingly expressed in the later stages of rind development (enlargement, coloring and maturity). Moreover, among the 28 genes, the percent expression of the most conserved RNL was the highest (75%), while that of the most relaxed CNL was lowest (32%). In addition, we found that the mean expression levels of the identified *NBS* genes at four different stages were 1.4, 1.5 and 2.2 FPKM in the seeds, flesh and rind, respectively (Figure 6B). Among the 66 expressed *NBS* genes, four genes were mainly expressed in the flesh, 16 in the seeds, and 46 in the rind (Figure 6C).

## Discussion

*NBS* genes are the largest disease *R* gene family that exists in all plants, and broad genome-wide analyses of this family have been carried out in recent years (Jones et al., 2016). Despite the existence of recent publications on the *A. trifoliata* genome (Huang et al., 2021), the corresponding genomic data files are still unavailable online. Therefore, a high quality *A. trafoliata* genome assembled and submitted by our group to NCBI was employed to identify and characterize *NBS* candidates (see detailed data in section “Materials and Methods”), including analyses of their numbers, structures, distribution, duplications and gene expression profiles.

Previous studies have shown that the number of *NBS* genes varies greatly among different species and usually reaches hundreds in a single plant species, to confer protection against diverse and rapidly evolving pathogens. There are over 2,000 *NBS* genes in the extremely large wheat genome, but these genes are extremely scarce in *C. papaya*, *Thellungiella salsuginea* and some orchids (Shao et al., 2016; Xue et al., 2019; Andersen et al., 2020). Here, we identified 73 *NBS* genes, accounting for only 0.3% of the 24,138 annotated genes of *A. trifoliata* (Table 2), which suggested that the number of *NBS* genes in its genome was small. In previous studies, some authors have suggested that a positive correlation exists between *NBS* gene number and genome size within five samples, where the number increases with increasing genome size (Goyal et al., 2020), while the others have inferred that there is no significant correlation between them also in five samples (Zhang et al., 2016). In this study, total 13 representative species including four basal eudicots, four core eudicots and five monocots (listed in Table 1), were employed to address this issue mainly because of the evolutionary relationship with *A. trifoliata*, available knowledge about NBS genes, and economic importance. The results showed a significant correlation coefficient (0.96) between *NBS* gene number and genome size among the 13 species; however, the value decreased to −0.14 after we removed wheat as the out-group (Table 1). Therefore, the correlation between *NBS* gene number and genome size could depend on both the size and components of the genome sample employed.

Despite the small number of *NBS* genes in *A. trifoliata*, the types of these genes are diverse, including 9 subclasses within 3 classes (Table 2), and these classes are distinct in composition (a 3:1 ratio of *nTNL*:*TNL*). Previous studies have shown that the *nTNL*:*TNL* ratio varies widely, ranging from 1:3 in *B. oleracea* (Liu et al., 2019) to an extremely high ratio of 18:1 in *Capsicum annuum* (Qian et al., 2017) among dicots, while monocots only contain the *nTNL* subclass because of the loss of the *TNL* subclass after the divergence of dicots and monocots (Qian et al., 2017). These large disparities in composition not only indicate that distinct *NBS* subgroups have experienced different evolutionary events, such as different types of genomic duplication and magnitudes of natural selection, but also provide key clues for establishing the relationships between speciation and the growth environment.

We found that there were markedly more *CNL* genes (68.5%) in *A. trifoliata* than *TNL* (26.0%) and *RNL* (5.5%) genes, which showed conserved evolution without large-scale expansion (Table 1). Moreover, the four *RNL*s formed an extremely conserved branch that did not deviate from the *CNLs* in Figure 5, and similarly low proportions of *RNLs* have been reported in some orchids (Xue et al., 2019) and *D. rotundata* (Zhang et al., 2020), in distinct contrast to the large numbers of *CNLs* and *TNLs*. A reasonable explanation for this phenomenon is that *RNLs* only assist other functional *NBS* genes as downstream genes with conserved functions, as reported for “Helper NBS-LRRs” (Bonardi et al., 2011; Collier et al., 2011). In fact, the number of *NBS* subfamilies retained in the genome usually depends on the genomic duplication type and the selection pressure exerted during evolutionary history (Mun et al., 2009). The analysis of the Ka/Ks ratio to explore the evolutionary difference between *CNL* and *TNL* showed that both *CNLs* and *TNLs* have experienced purifying selection, and the significantly lower Ka/Ks value of *CNLs* than *TNLs* at the *p* = 0.01 level (Supplementary Table 5) demonstrated that greater selective constraints and purifying selection in the *CNL* group and relaxation of purifying selection in the *TNL* group might have resulted in ancient diversification of *NBS* genes (Mun et al., 2009).

As the primary source of raw genetic material, WGD is widely involved in plant evolutionary processes and is especially common in angiosperms (Akoz and Nordborg, 2019), but only two *NBS* genes from the ancestral WGD event survived in *A. trifoliata* (Supplementary Table 1). This was probably because the synteny of *NBS* genes resulting from WGD was destroyed in the process of long-term evolution or because the number of *NBS* genes might have been reduced after WGD via rapid species-specific gene loss (Die et al., 2018a; Song et al., 2019). Recently, some studies have suggested that tandem and dispersed duplication events could be a major driving force of *NBS* gene expansion (Baumgarten et al., 2003; Qian et al., 2017); however, the main duplications in each subclass of *NBS* are still unclear. In the present study, we found that in the *A. trifoliata* genome, *CNLs* were mainly expanded by the dispersal of individual or small groups of duplicated genes to unlinked loci, while *TNLs* were mainly expanded by tandem duplication, which produced closely related genes in the same family that clustered together (Supplementary Table 1; Leister, 2004). Additionally, the chromosomal distribution of *NBS* genes in the *A. trifoliata* genome (Figure 2) agreed well with the differences in gene expansion, which could also explain the greater number of *CNLs* relative to *TNLs*.

The other interesting characteristic involved the relationship between exon number and gene length in the *A*. *trifoliata* genome, where a significant positive relationship existed between exon number and gene length among the 73 *NBS* genes (Table 2). We further found that *TNLs* showed larger values than *CNLs* for both exon number and gene length in all other six dicots on our list (Supplementary Table 7), including *Raphanus sativus* (Ma et al., 2021), *A. thaliana*, *B. oleraces*, *B. rapa* (Mun et al., 2009), *B. napus* (Alamery et al., 2018), *Helianthus annuus* (Neupane et al., 2018), and *Manihot esculenta* (Lozano et al., 2015). All results from the above species indicated that independent gains or losses of exons occurred in distinct *NBS* subclasses, and the greater exon number and longer gene lengths of *TNLs* could result from a more ancient evolutionary history than *CNLs*, which evolved more recently (Meyers et al., 2003).

Structurally, the NBS domains of the characterized *R* genes shared eight common conserved motifs (Meyers et al., 2003). Through MEME analysis, we found that 45.2% of the identified genes contained all eight motifs, and P-loop (66), GLPL (68) and kinase-2 (70) motifs were the most common ones (Supplementary Table 3). Both the P-loop and kinase-2 motifs function in ATP/GTP binding (Traut, 1994; Meyers et al., 2003), and the existence of these typically conserved NBS motifs in *R* proteins is essential for protein function. Although the specific function of GLPL is still poorly understood, its importance in disease resistance has been demonstrated by site mutation analysis in the context of flax rust (Dodds et al., 2001). Thus, the higher the frequency distribution of a motif is, the more conserved it is. In addition, the order of these eight motifs was shown to be relatively inflexible, and only two (*AtNBS19* and *AtNBS63*) of the 73 identified *NBS* genes in *A*. *trifoliata* actually showed an order change (Figure 1 and Supplementary Table 2). We inferred that the conserved order of these motifs could be more important than the sequences themselves.

We further observed that the last residue of the kinase-2 motif exhibited the main difference between *TNL* and *nTNL*: 88.9% of *nTNL*s contained W as the last residue of the kinase-2 motif, while it was replaced with D in 68.4% of *TNL*s (Supplementary Table 3), which was consistent with previous works (Meyers et al., 2003; Die et al., 2018b). Initial reports suggested that the conserved aspartate of kinase-2 is indispensable for the concentration-dependent binging of Mg^2+^ to ATP, which is essential for its function in plant defense signaling (Tameling et al., 2002). Thus, we inferred that the TNL proteins with more conserved aspartate residues may be more functional ATP-binding proteins with ATPase activity.

Homologous proteins with sequence similarity usually retain similar functions in evolution, and consequently we aimed to select some key and well known *NBS* resistance genes from other species, which was conducive to the identification of close functional homologs in *A. trifoliata* (Lozano et al., 2015). Then, the TNL (Figure 4) and CNL (Figure 5) trees were separately reconstructed by adding reference *TNLs* and *CNL*s/*RNL*s. Many *TNL* candidates clustered with functional genes for resistance to bacterial or fungal root rot diseases in TNL-B, and five *CNL* candidates clustered with the *Dm3* gene for resistance to downy mildew in clade CNL-2b under 100% bootstrap support, while only *CNL* candidates were included in clade CNL-4, indicating that these genes might provide resistance to unknown *A. trifoliata* pathogens or play a role in non-host resistance responses. The widespread *A. trifoliata NBS* genes in the phylogenetic trees suggested that the different *NBS* subclasses could provide broad resistance to various pathogen species, while a few *NBS* genes would confer resistance to diverse races of the same pathogen. Furthermore, *CNLs* showed a higher level of topological complexity than *TNLs* in Figure 3, which might be helpful for broadening the resistance spectrum to achieve a broadly suitable distribution and could explain the long evolutionary history of this basal eudicot (Sun et al., 2016; Qian et al., 2017).

Comparative transcriptomic analysis can provide important insight into gene structures, functions and application prospects (Lowe et al., 2017). In the present study, we found that 90.4% of *NBS* genes were expressed in various fruit tissues at different developmental stages, and most of them were expressed at low levels (Figure 6A), which indicated that they have broad functions under field conditions. The relatively large number of *NBS* genes (46 of 66) that were mainly expressed in the rind at higher expression levels, especially in later stages, also indicated that most of these genes functionally conferred broad horizontal resistance to various pathogens in the field because only the rind tissue of *A. trifoliata* is exposed to pathogens. In addition, rind is usually considered the first barrier to pathogen infection, and hence the higher *NBS* gene expression compared with both flesh and seed in the later stages is reasonable. Only *AtNBS51* (*RNL*) was identified as a gene with intermediate or high expression, and 27 NBS genes were identified as genes with low expression (Supplementary Table 6), which indicates that no pathogen can escape the defense exerted by a few *NBS* genes under a given condition. In addition, the high proportion (75%) of stably expressed *RNLs* despite the small proportion (5.5%) of *RNLs* among all 73 genes further demonstrated that with the help of *RNLs*, many *CNLs* and *TNLs* of *A. trifoliata* can confer broad resistance to various pathogen species and/or different races of the same species. Together, all evidence suggests that the small number of NBSs with structural diversification in *A. trifoliata* can provide defense against attacks by all types of pathogens that may be encountered. From the perspective of plant pathology, this provides a reasonable explanation for the wide distribution of wild *A. trifoliata* in various geographical and ecological regions and its continued healthy growth over many years.

In conclusion, only 73 non-redundant *NBS* genes were identified in the *A. trifoliata* genome in this work, but they showed high diversity, including three classes (*CNL*, *TNL* and *RNL*) with nine subclasses. Evolutionarily, although almost all *NBS* genes were retained by purifying selection, the duplication style was different: *CNLs* were mainly expanded by dispersed duplication, while *TNLs* were mainly expanded by tandem duplication. Structurally, the order of the eight conserved motifs in the NBS domain was more conserved than their amino acid sequences. Functionally, differential expression analysis showed that many of *NBS* genes could play an actual role in fighting pathogens by conferring broad-spectrum resistance, which may have contributed to the genetic basis favoring adaptation to various environments during the evolutionary of *A. trifoliata*. Therefore, this study paves the way for improving disease resistance during the domestication of *A. trifoliata* and further enriches the available information about *NBS* genes, especially in basal eudicots.

## Data Availability Statement

The datasets presented in this study can be found in online repositories. The names of the repository/repositories and accession number(s) can be found in the article/Supplementary Material.

## Author Contributions

XY and PL conceived and designed the project. SZ obtained the data. XY analyzed the data and wrote the manuscript. CC, WC, HuY, HaY, JG, FT, TR, JS, MZ, and PF participated in the data analysis and discussion. PL revised the manuscript. All authors contributed to the discussion of the results, reviewed the manuscript and approved the final article.

## Conflict of Interest

WC and HaY were employed by the company Sichuan Akebiatrifoliata Biotechnology Co., Ltd. The remaining authors declare that the research was conducted in the absence of any commercial or financial relationships that could be construed as a potential conflict of interest.

## Publisher’s Note

All claims expressed in this article are solely those of the authors and do not necessarily represent those of their affiliated organizations, or those of the publisher, the editors and the reviewers. Any product that may be evaluated in this article, or claim that may be made by its manufacturer, is not guaranteed or endorsed by the publisher.

## Footnotes

1
https://www.ncbi.nlm.nih.gov/bioproject/?term=PRJNA671772

2
https://blast.ncbi.nlm.nih.gov/Blast.cgi

3
https://www.ebi.ac.uk/Tools/hmmer/

4
http://pfam.sanger.ac.uk/

5
https://www.ncbi.nlm.nih.gov/Structure/cdd/

6
http://coiledcoils.chm.bris.ac.uk/Scorer/

7
http://meme-suite.org/tools/meme

8
https://github.com/CJ-Chen/TBtools

9
https://github.com/rambaut/figtree/releases

10
https://www.ncbi.nlm.nih.gov/biosample

## Supplementary Material

Supplementary Figure 1 The complete tree of CNL subclass of *A. trifoliata* NBS proteins.

Supplementary Table 1 Detailed features of identified NBS genes in *A. trifoliata*.

Supplementary Table 2 Differential arrangement of conserved motifs in NBS domain.

Supplementary Table 3 Discovered Motifs Statistics of NBS domain in *A. trifoliata* NBS genes.

Supplementary Table 4 Reference NBS genes identified from other species for reconstructed phylogenetic trees.

Supplementary Table 5 Ka Ks and Ka/Ks values of *A. trifoliata* NBS gene pairs.

Supplementary Table 6 NBS genes Expression profile of the three *A. trifoliata* tissues and four development stages.

Supplementary Table 7 *TNLs* and *CNLs* for both exon number and gene length in 7 dicots.

## References

[B1] Akoz G. Nordborg M. (2019). The Aquilegia genome reveals a hybrid origin of core eudicots. *Genome Biol*. 20:256. 10.1186/s13059-019-1888-8 31779695 PMC6883705

[B2] Alamery S. Tirnaz S. Bayer P. Tollenaere R. Chaloub B. Edwards D. (2018). Genome-wide identification and comparative analysis of NBS-LRR resistance genes in Brassica napus. *Crop Pasture Sci*. 69 79–93. 10.1071/cp17214

[B3] Ameline-Torregrosa C. Wang B. B. O’Bleness M. S. Deshpande S. Zhu H. Roe B. (2008). Identification and characterization of nucleotide-binding site-leucine-rich repeat genes in the model plant Medicago truncatula. *Plant Physiol*. 146 5–21. 10.1104/pp.107.104588 17981990 PMC2230567

[B4] Andersen E. J. Ali S. Byamukama E. Yen Y. Nepal M. P. (2018). Disease Resistance Mechanisms in Plants. *Genes* 9:339. 10.3390/genes9070339 29973557 PMC6071103

[B5] Andersen E. J. Nepal M. P. Purintun J. M. Nelson D. Mermigka G. Sarris P. F. (2020). Wheat Disease Resistance Genes and Their Diversification Through Integrated Domain Fusions. *Front. Genet*. 11:898. 10.3389/fgene.2020.00898 32849852 PMC7422411

[B6] Baumgarten A. Cannon S. Spangler R. May G. (2003). Genome-level evolution of resistance genes in Arabidopsis thaliana. *Genetics* 165 309–319. 10.1023/A:1024446910161 14504238 PMC1462749

[B7] Bent A. F. (1996). Plant Disease Resistance Genes: function Meets Structure. *Plant Cell* 8 1757–1771. 10.1105/tpc.8.10.1757 12239361 PMC161313

[B8] Bent A. F. Kunkel B. N. Dahlbeck D. Brown K. L. Schmidt R. Giraudat J. (1994). RPS2 of Arabidopsis thaliana: a leucine-rich repeat class of plant disease resistance genes. *Science* 265 1856–1860. 10.1126/science.8091210 8091210

[B9] Bonardi V. Tang S. Stallmann A. Roberts M. Cherkis K. Dangl J. L. (2011). Expanded functions for a family of plant intracellular immune receptors beyond specific recognition of pathogen effectors. *Proc. Natl. Acad. Sci. U. S. A*. 108 16463–16468. 10.1073/pnas.1113726108 21911370 PMC3182704

[B10] Chen C. Chen H. Zhang Y. Thomas H. R. Frank M. H. He Y. (2020). TBtools: an Integrative Toolkit Developed for Interactive Analyses of Big Biological Data. *Mol. Plant* 13 1194–1202. 10.1016/j.molp.2020.06.009 32585190

[B11] Chen W. Zhong S. Chen H. Luo P. (2017). Studies about the Utilization and Development of Akebia trifoliata and Its Targeted Poverty Alleviation Strategies —A Case Study in Shimian County, Sichuan Province. *Chin. Wild Plant Resour*. 36 71–74. 10.3969/j.issn.1006-9690.2017.05.016

[B12] Cheng S. Ke J. Tan L. T. Medison R. G. Liang P. Gong T. (2020). First Report of Leaf Spot on Akebia trifoliata Caused by Phytophthora nicotianae in China. *Plant Dis*. 10.1094/PDIS-06-20-1243-PDN [Online ahead of print]. 32976077

[B13] Collier S. M. Hamel L. P. Moffett P. (2011). Cell death mediated by the N-terminal domains of a unique and highly conserved class of NB-LRR protein. *Mol. Plant Microbe Interact*. 24 918–931. 10.1094/MPMI-03-11-0050 21501087

[B14] Collier S. M. Moffett P. (2009). NB-LRRs work a “bait and switch” on pathogens. *Trends Plant Sci*. 14 521–529. 10.1016/j.tplants.2009.08.001 19720556

[B15] Die J. V. Castro P. Millan T. Gil J. (2018a). Segmental and Tandem Duplications Driving the Recent NBS-LRR Gene Expansion in the Asparagus Genome. *Genes* 9:568. 10.3390/genes9120568 30477134 PMC6316259

[B16] Die J. V. Roman B. Qi X. Rowland L. J. (2018b). Genome-scale examination of NBS-encoding genes in blueberry. *Sci. Rep*. 8:3429. 10.1038/s41598-018-21738-7 29467425 PMC5821885

[B17] Dodds P. N. Lawrence G. J. Ellis J. G. (2001). Six amino acid changes confined to the leucine-rich repeat beta-strand/beta-turn motif determine the difference between the P and P2 rust resistance specificities in flax. *Plant Cell* 13 163–178. 10.1105/tpc.13.1.163 11158537 PMC102207

[B18] Dudley W. N. Benuzillo J. G. Carrico M. S. (2004). SPSS and SAS programming for the testing of mediation models. *Nurs. Res*. 53 59–62. 10.1097/00006199-200401000-00009 14726778

[B19] Garibaldi A. Bertetti D. Gullino M. L. (2004). First Report of Powdery Mildew (Oidium sp.) on Akebia quinata in Italy. *Plant Dis.* 88:682. 10.1094/pdis.2004.88.6.682d 30812603

[B20] Goyal N. Bhatia G. Sharma S. Garewal N. Upadhyay A. Upadhyay S. K. (2020). Genome-wide characterization revealed role of NBS-LRR genes during powdery mildew infection in Vitis vinifera. *Genomics* 112 312–322. 10.1016/j.ygeno.2019.02.011 30802599

[B21] Hoang D. T. Chernomor O. von Haeseler A. Minh B. Q. Vinh L. S. (2018). UFBoot2: improving the Ultrafast Bootstrap Approximation. *Mol. Biol. Evol*. 35 518–522. 10.1093/molbev/msx281 29077904 PMC5850222

[B22] Hong X. Chen S. Wang L. Liu B. Yang Y. Tang X. (2021). First report of Nigrospora sphaerica causing fruit dried-shrink disease in Akebia trifoliata from China. *Plant Dis.* 10.1094/PDIS-11-20-2471-PDN [Online ahead of print]. 35130035

[B23] Huang C. L. Hwang S. Y. Chiang Y. C. Lin T. P. (2008). Molecular evolution of the Pi-ta gene resistant to rice blast in wild rice (Oryza rufipogon). *Genetics* 179 1527–1538. 10.1534/genetics.108.089805 18622033 PMC2475752

[B24] Huang H. Liang J. Tan Q. Ou L. Li X. Zhong C. (2021). Insights into triterpene synthesis and unsaturated fatty-acid accumulation provided by chromosomal-level genome analysis of Akebia trifoliata subsp. australis. *Hortic. Res*. 8:33. 10.1038/s41438-020-00458-y 33518712 PMC7848005

[B25] Hurst L. D. (2002). The Ka/Ks ratio: diagnosing the form of sequence evolution. *Trends Genet*. 18:486. 10.1016/s0168-9525(02)02722-1 12175810

[B26] Jones J. D. Vance R. E. Dangl J. L. (2016). Intracellular innate immune surveillance devices in plants and animals. *Science* 354:aaf6395. 10.1126/science.aaf6395 27934708

[B27] Jupe F. Pritchard L. Etherington G. J. Mackenzie K. Cock P. J. Wright F. (2012). Identification and localisation of the NB-LRR gene family within the potato genome. *BMC Genomics* 13:75. 10.1186/1471-2164-13-75 22336098 PMC3297505

[B28] Kalyaanamoorthy S. Minh B. Q. Wong T. K. F. von Haeseler A. Jermiin L. S. (2017). ModelFinder: fast model selection for accurate phylogenetic estimates. *Nat. Methods* 14 587–589. 10.1038/nmeth.4285 28481363 PMC5453245

[B29] Kim D. Paggi J. M. Park C. Bennett C. Salzberg S. L. (2019). Graph-based genome alignment and genotyping with HISAT2 and HISAT-genotype. *Nat. Biotechnol*. 37 907–915. 10.1038/s41587-019-0201-4 31375807 PMC7605509

[B30] Kourelis J. van der Hoorn R. A. L. (2018). Defended to the Nines: 25 Years of Resistance Gene Cloning Identifies Nine Mechanisms for R Protein Function. *Plant Cell* 30 285–299. 10.1105/tpc.17.00579 29382771 PMC5868693

[B31] Kumar S. Stecher G. Li M. Knyaz C. Tamura K. (2018). MEGA X: molecular Evolutionary Genetics Analysis across Computing Platforms. *Mol. Biol. Evol*. 35 1547–1549. 10.1093/molbev/msy096 29722887 PMC5967553

[B32] Leister D. (2004). Tandem and segmental gene duplication and recombination in the evolution of plant disease resistance genes. *Trends Genet*. 20 116–122. 10.1016/j.tig.2004.01.007 15049302

[B33] Li H. Handsaker B. Wysoker A. Fennell T. Ruan J. Homer N. (2009). The Sequence Alignment/Map format and SAMtools. *Bioinformatics* 25 2078–2079. 10.1093/bioinformatics/btp352 19505943 PMC2723002

[B34] Li J. Ding J. Zhang W. Zhang Y. Tang P. Chen J. Q. (2010). Unique evolutionary pattern of numbers of gramineous NBS-LRR genes. *Mol. Genet. Genomics* 283 427–438. 10.1007/s00438-010-0527-6 20217430

[B35] Li L. Yao X. H. Zhong C. H. Chen X. Z. Huang H. W. (2010). Akebia: a Potential New Fruit Crop in China. *Hortscience* 45 4–10. 10.21273/Hortsci.45.1.4

[B36] Liu G. Y. Ma S. C. Zheng J. Zhang J. Lin R. C. (2007). Two new triterpenoid saponins from Akebia quinata (Thunb.) decne. *J. Integr. Plant Biol*. 49 196–201. 10.1111/j.1744-7909.2007.00362.x

[B37] Liu S. Liu Y. Yang X. Tong C. Edwards D. Parkin I. A. (2014). The Brassica oleracea genome reveals the asymmetrical evolution of polyploid genomes. *Nat. Commun*. 5:3930. 10.1038/ncomms4930 24852848 PMC4279128

[B38] Liu Z. Xie J. Wang H. Zhong X. Li H. Yu J. (2019). Identification and expression profiling analysis of NBS-LRR genes involved in Fusarium oxysporum f.sp. conglutinans resistance in cabbage. *3 Biotech* 9:202. 10.1007/s13205-019-1714-8 31065502 PMC6500516

[B39] Lowe R. Shirley N. Bleackley M. Dolan S. Shafee T. (2017). Transcriptomics technologies. *PLoS Comput. Biol*. 13:e1005457. 10.1371/journal.pcbi.1005457 28545146 PMC5436640

[B40] Lozano R. Hamblin M. T. Prochnik S. Jannink J. L. (2015). Identification and distribution of the NBS-LRR gene family in the Cassava genome. *BMC Genomics* 16:360. 10.1186/s12864-015-1554-9 25948536 PMC4422547

[B41] Ma J. Zhang C. Yang D. Xie Y. Sun H. (2020). Research Progress of Sweet Potato Virus Disease in China. *J. Hebei Agric. Sci*. 24 51–56. 10.12148/hbnykx.20200051

[B42] Ma Y. Chhapekar S. S. Lu L. Oh S. Singh S. Kim C. S. (2021). Genome-wide identification and characterization of NBS-encoding genes in Raphanus sativus L. and their roles related to Fusarium oxysporum resistance. *BMC Plant Biol*. 21:47. 10.1186/s12870-020-02803-8 33461498 PMC7814608

[B43] Meyers B. C. Kozik A. Griego A. Kuang H. Michelmore R. W. (2003). Genome-wide analysis of NBS-LRR-encoding genes in Arabidopsis. *Plant Cell* 15 809–834. 10.1105/tpc.009308 12671079 PMC152331

[B44] Mun J. H. Yu H. J. Park S. Park B. S. (2009). Genome-wide identification of NBS-encoding resistance genes in Brassica rapa. *Mol. Genet. Genomics* 282 617–631. 10.1007/s00438-009-0492-0 19838736 PMC2777221

[B45] Nepal M. P. Andersen E. J. Neupane S. Benson B. V. (2017). Comparative Genomics of Non-TNL Disease Resistance Genes from Six Plant Species. *Genes* 8:249. 10.3390/genes8100249 28973974 PMC5664099

[B46] Neupane S. Andersen E. J. Neupane A. Nepal M. P. (2018). Genome-Wide Identification of NBS-Encoding Resistance Genes in Sunflower (Helianthus annuus L.). *Genes* 9:384. 10.3390/genes9080384 30061549 PMC6115920

[B47] Nguyen L. T. Schmidt H. A. von Haeseler A. Minh B. Q. (2015). IQ-TREE: a fast and effective stochastic algorithm for estimating maximum-likelihood phylogenies. *Mol. Biol. Evol*. 32 268–274. 10.1093/molbev/msu300 25371430 PMC4271533

[B48] Pan H. Deng L. Feng D. Zhong C. Li L. (2020). First Report of Anthracnose Caused by Colletotrichum gloeosporioides on Akebia trifoliata in China. *Plant Dis.* 10.1094/PDIS-07-20-1525-PDN [Online ahead of print]. 32915120

[B49] Porter B. W. Paidi M. Ming R. Alam M. Nishijima W. T. Zhu Y. J. (2009). Genome-wide analysis of Carica papaya reveals a small NBS resistance gene family. *Mol. Genet. Genomics* 281 609–626. 10.1007/s00438-009-0434-x 19263082

[B50] Qian L. H. Zhou G. C. Sun X. Q. Lei Z. Zhang Y. M. Xue J. Y. (2017). Distinct Patterns of Gene Gain and Loss: diverse Evolutionary Modes of NBS-Encoding Genes in Three Solanaceae Crop Species. *G3* 7 1577–1585. 10.1534/g3.117.040485 28364035 PMC5427506

[B51] Scortichini M. Marcelletti S. Ferrante P. Petriccione M. Firrao G. (2012). *Pseudomonas* syringae pv. actinidiae: a re-emerging, multi-faceted, pandemic pathogen. *Mol. Plant Pathol*. 13 631–640. 10.1111/j.1364-3703.2012.00788.x 22353258 PMC6638780

[B52] Shao Z. Q. Xue J. Y. Wang Q. Wang B. Chen J. Q. (2019). Revisiting the Origin of Plant NBS-LRR Genes. *Trends Plant Sci*. 24 9–12. 10.1016/j.tplants.2018.10.015 30446304

[B53] Shao Z. Q. Xue J. Y. Wu P. Zhang Y. M. Wu Y. Hang Y. Y. (2016). Large-Scale Analyses of Angiosperm Nucleotide-Binding Site-Leucine-Rich Repeat Genes Reveal Three Anciently Diverged Classes with Distinct Evolutionary Patterns. *Plant Physiol*. 170 2095–2109. 10.1104/pp.15.01487 26839128 PMC4825152

[B54] Song H. Guo Z. Hu X. Qian L. Miao F. Zhang X. (2019). Evolutionary balance between LRR domain loss and young NBS-LRR genes production governs disease resistance in *Arachis hypogaea* cv. Tifrunner. *BMC Genomics* 20:844. 10.1186/s12864-019-6212-1 31722670 PMC6852974

[B55] Sun Y. Moore M. J. Zhang S. Soltis P. S. Soltis D. E. Zhao T. (2016). Phylogenomic and structural analyses of 18 complete plastomes across nearly all families of early-diverging eudicots, including an angiosperm-wide analysis of IR gene content evolution. *Mol. Phylogenet. Evol*. 96 93–101. 10.1016/j.ympev.2015.12.006 26724406

[B56] Tameling W. I. Elzinga S. D. Darmin P. S. Vossen J. H. Takken F. L. Haring M. A. (2002). The tomato R gene products I-2 and MI-1 are functional ATP binding proteins with ATPase activity. *Plant Cell* 14 2929–2939. 10.1105/tpc.005793 12417711 PMC152737

[B57] Traut T. W. (1994). The functions and consensus motifs of nine types of peptide segments that form different types of nucleotide-binding sites. *Eur. J. Biochem*. 222 9–19. 10.1111/j.1432-1033.1994.tb18835.x 8200357

[B58] Tuskan G. A. Difazio S. Jansson S. Bohlmann J. Grigoriev I. Hellsten U. (2006). The genome of black cottonwood, Populus trichocarpa (Torr. & Gray). *Science* 313 1596–1604. 10.1126/science.1128691 16973872

[B59] Wang D. Zhang Y. Zhang Z. Zhu J. Yu J. (2010). KaKs_Calculator 2.0: a toolkit incorporating gamma-series methods and sliding window strategies. *Genomics Proteomics Bioinformatics* 8 77–80. 10.1016/s1672-0229(10)60008-3 20451164 PMC5054116

[B60] Wang W. Zhang Y. Wen Y. Berkey R. Ma X. Pan Z. (2013). A comprehensive mutational analysis of the Arabidopsis resistance protein RPW8.2 reveals key amino acids for defense activation and protein targeting. *Plant Cell* 25 4242–4261. 10.1105/tpc.113.117226 24151293 PMC3877822

[B61] Wang Y. Tang H. Debarry J. D. Tan X. Li J. Wang X. (2012). MCScanX: a toolkit for detection and evolutionary analysis of gene synteny and collinearity. *Nucleic Acids Res*. 40:e49. 10.1093/nar/gkr1293 22217600 PMC3326336

[B62] Xiao S. Ellwood S. Calis O. Patrick E. Li T. Coleman M. (2001). Broad-spectrum mildew resistance in Arabidopsis thaliana mediated by RPW8. *Science* 291 118–120. 10.1126/science.291.5501.118 11141561

[B63] Xue J. Y. Zhao T. Liu Y. Liu Y. Zhang Y. X. Zhang G. Q. (2019). Genome- Wide Analysis of the Nucleotide Binding Site Leucine-Rich Repeat Genes of Four Orchids Revealed Extremely Low Numbers of Disease Resistance Genes. *Front. Genet*. 10:1286. 10.3389/fgene.2019.01286 31998358 PMC6960632

[B64] Ye Y. F. Jiang N. Fu G. Liu W. Hu F. Y. Liu L. H. (2013). First Report of Corynespora cassiicola Causing Leaf Spot on Akebia trifoliate. *Plant Dis*. 97:1659. 10.1094/PDIS-04-13-0454-PDN 30716840

[B65] Zhang Y. M. Chen M. Sun L. Wang Y. Yin J. Liu J. (2020). Genome-Wide Identification and Evolutionary Analysis of NBS-LRR Genes From Dioscorea rotundata. *Front. Genet*. 11:484. 10.3389/fgene.2020.00484 32457809 PMC7224235

[B66] Zhang Y. M. Shao Z. Q. Wang Q. Hang Y. Y. Xue J. Y. Wang B. (2016). Uncovering the dynamic evolution of nucleotide-binding site-leucine-rich repeat (NBS-LRR) genes in Brassicaceae. *J. Integr. Plant Biol*. 58 165–177. 10.1111/jipb.12365 25926337

[B67] Zhang Z. Li X. Liu Y. (2015). Biological characteristics and biological control of leaf spot disease pathogen on Akebia trifoliata. *J. shaanxi Normal Univ.* 43 61–66. 10.15983/j.cnki.jsnu.2015.04.353

[B68] Zhao Y. Weng Q. Song J. Ma H. Yuan J. Dong Z. (2016). Bioinformatics Analysis of NBS-LRR Encoding Resistance Genes in Setaria italica. *Biochem. Genet*. 54 232–248. 10.1007/s10528-016-9715-3 26846709

[B69] Zhou J. Zhou Z. (2017). Anthrax of apple and its control technology. *Shanxi Fruits* 4 76–77. 10.16010/j.cnki.14-1127/s.2019.04.028

[B70] Zhou T. Wang Y. Chen J. Q. Araki H. Jing Z. Jiang K. (2004). Genome-wide identification of NBS genes in japonica rice reveals significant expansion of divergent non-TIR NBS-LRR genes. *Mol. Genet. Genomics* 271 402–415. 10.1007/s00438-004-0990-z 15014983

