# Supplementary figures and images for: Identification and Characterization of NBS Resistance Genes in Akebia trifoliata
Source: Front Plant Sci. 2021 Oct 29;12:758559. doi: 10.3389/fpls.2021.758559 (PMC8585750; doi:10.3389/fpls.2021.758559)

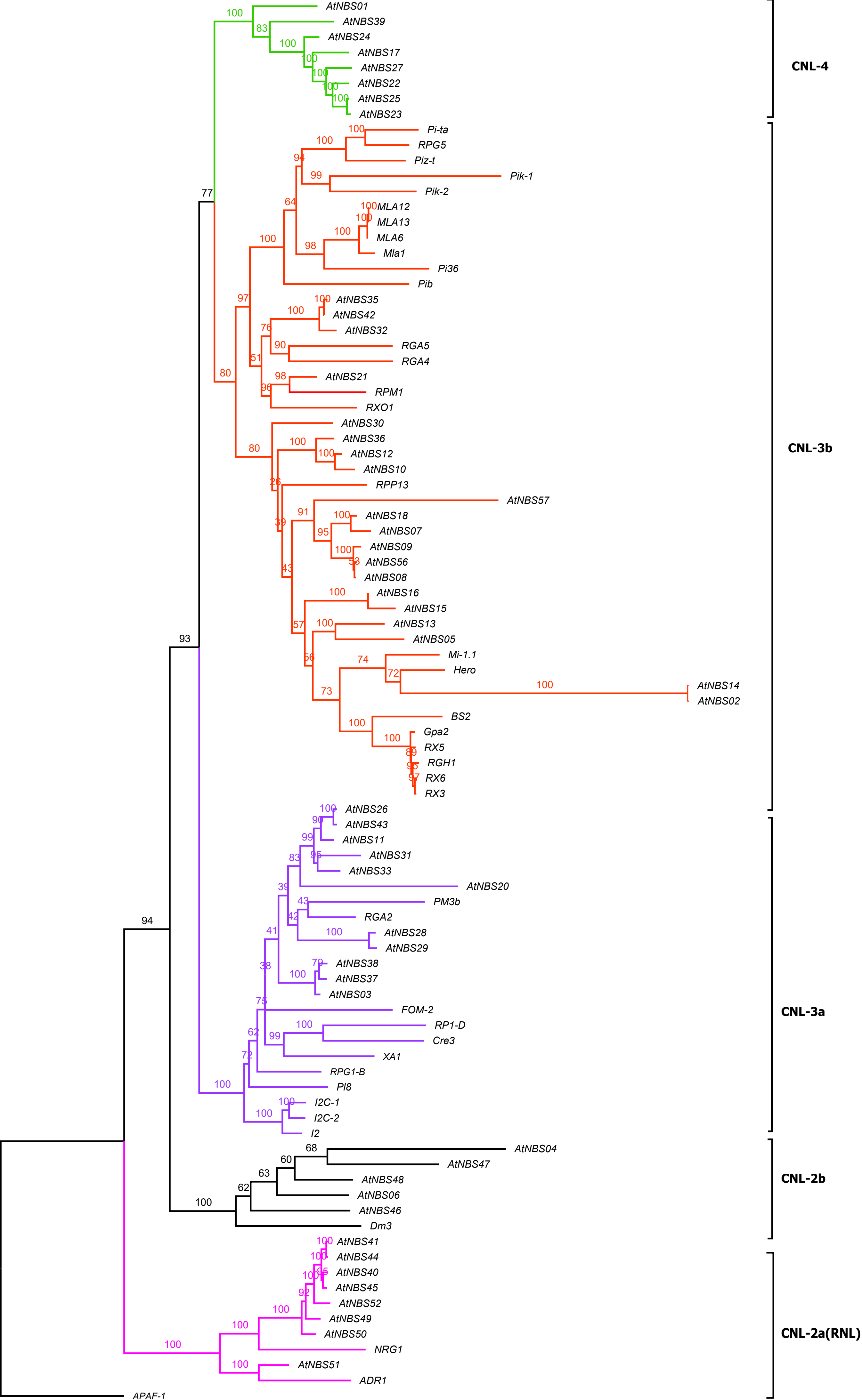

Supplement: Supplementary Figure 1 — The complete tree of CNL subclass of A. trifoliata NBS proteins. [file Data_Sheet_1.PDF]
